# Rabies in a dog commercially imported into Germany from Russia, 2026

**DOI:** 10.2807/1560-7917.ES.2026.31.19.2600372

**Published:** 2026-05-14

**Authors:** Conrad M Freuling, Lisa Rogoll, Sten Calvelage, Dirk Höper, Dmitry Ushakov, Laura Rzepa, Laura Schmid, Stefan Finke, Irene Klingelhöfer, Gudrun Larres, Christin Krüger, Dirk Steinhauer, Silvia Eisch-Wolf, Martin Dehler, Carola Sauter-Louis, Thomas Mueller

**Affiliations:** 1 Friedrich-Loeffler-Institute (FLI), Institute of Molecular Virology and Cell Biology, Greifswald-Insel Riems, Germany; 2 Friedrich-Loeffler-Institute (FLI), Institute of Epidemiology, Greifswald-Insel Riems, Germany; 3 Friedrich-Loeffler-Institute (FLI), Institute of Diagnostic Virology, Greifswald-Insel Riems, Germany; 4 Institute for Animal Disease Diagnostics, State Investigation Office Rhineland Palatinate, Koblenz, Germany; 5 Ministry for Climate Protection, Environment, Energy and Mobility, Division of Animal Health and Animal Diseases, Mainz, Germany; 6 Administrative District Rhein-Pfalz, Public Health Department, Ludwigshafen, Germany

**Keywords:** rabies, dog, import, post exposure prophylaxis, lyssavirus

## Abstract

Although rabies has been eliminated from most European Union countries, the risk of reintroduction persists through the movement of infected animals, particularly via non-compliant imports. We describe a case of rabies in a dog imported commercially from Russia to Germany in 2025. The infection was confirmed in February 2026, with phylogenetic analysis linking the virus to the Cosmopolitan lineage (cluster C1b). Investigations revealed documentation inconsistencies, including suspected falsified vaccination records, highlighting the need for strengthened import controls.

Rabies caused by classical rabies virus (RABV) is eliminated from most European Union (EU) countries [1,2]. Nevertheless, the EU remains at risk of reintroduction through the movement of infected animals from rabies-endemic countries [3].To prevent such introductions, the EU has established harmonised requirements for non-commercial movement of pets under Regulations (EU) No 576/2013. If followed, these measures are highly effective in mitigating the risk of rabies incursion [3-5]. Here we report on a rabies case from a commercial importation from Russia.

## Case description

In February 2026, rabies was confirmed in a female dog in Rhineland-Palatinate, Germany. The dog had been imported from Russia in November 2025. On 22 January 2026, the dog developed anorexia and was admitted to a veterinary clinic, where suspicion of a foreign body was basis for a surgical intervention. While still hospitalised, the dog lost two primary (deciduous) teeth after aggressively biting the cage bars. Aggressive behaviour continued at home, and due to the suspicion of rabies, the animal was placed in quarantine on 25 January, where she died on the night of 26 January 2026. Contact tracing identified eight exposed persons (see public health measures).

## Virological and genomic investigations

Initial direct fluorescent antibody testing (FAT) on brain material from the dog by the State Laboratory of Rhineland-Palatinate yielded negative results on 28 January. Virus isolation from brain material in mouse neuroblastoma cell culture subsequently confirmed rabies, which was further verified by RT-qPCR [6] on 10 February 2026. The case was immediately reported through the online platform interactive Rapid Alert System for Food and Feed (iRASFF) and the Animal Disease Information System (ADIS) to the European Commission and automatically notified to the World Organisation for Animal Health (WOAH) via WAHIS.

Both the initial negative FAT result and the positive RT-qPCR findings were confirmed at the National Reference Laboratory (NRL) for Rabies at the Friedrich-Loeffler-Institute (FLI). Confocal laser scan image analyses demonstrated rabies antigen stained by both anti-N and anti-P antibodies, as presented in Supplementary material.

**Figure 1:**
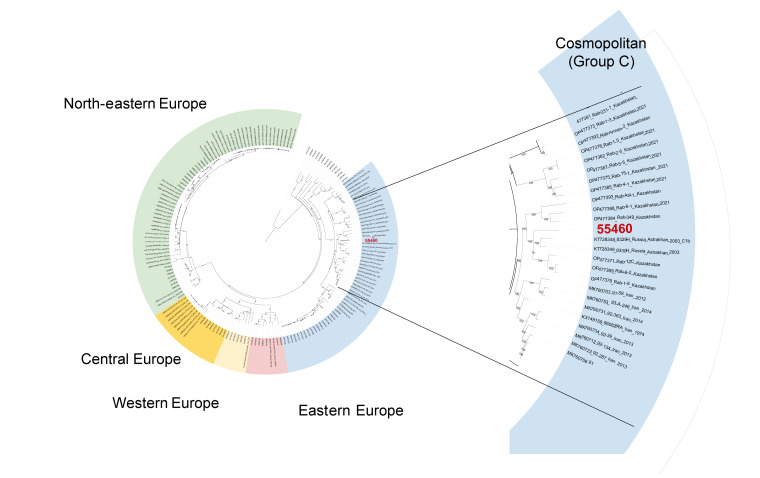
Maximum likelihood phylogenetic tree of different full-length genome sequences of rabies virus, collected between 1972 and 2024 (n = 149)

Full genome sequence analysis revealed that the rabies virus isolated from the imported dog (FLI laboratory ID: 55460) belonged to the Cosmopolitan lineage of RABV, showing the highest identity with RABV sequences from cluster C1b (Figure 1), a cluster associated with RABV from south-eastern Russia, Kazakhstan and China [7].

The isolate from the current case report is marked red in the figure. The phylogenetic tree was realised with IQTree (best fit model according to ModelFinder: GTR + F + I + G4).

Rabies virus lineage nomenclature was used as previously described [18]. Methodology for sequence generation and the details for the sequences used in the phylogenetic tree are included in Supplementary material.

## Epidemiolocal investigations

The dog was part of a consignment of 24 pets (22 dogs and 2 cats) imported by a Russian animal welfare organisation via a Latvian border control post. According to the accompanying documentation (Animal Health Certificate and EU Pet Passport), the dog was reportedly born on 5 May 2025, microchipped, vaccinated against rabies in July 2025, and subjected to rabies antibody titration testing before entry into the EU, with a reported titre of 2.0 IU/ml in September 2025. Thus, the animal appeared to meet all requirements for commercial importation into the EU under Commission Implementing Regulation (EU) 2021/403 and Commission Delegated Regulation (EU) 2020/692. However, post-mortem examination estimated the dog’s age at 4–6 months at the time of death, suggesting a birth date no earlier than end of July or August 2025. This latter date was also reported in a chat group of the charity organisation, indicating that the documentation presented was likely fraudulent. This is corroborated by the forensic assessment of the vaccine manufacturer, that the vaccine label in the pet passport was not authentic.

Further investigations revealed that the transport crossed the Belarusian border into Latvia on 21 November 2025 at a veterinary border control post, where documentary, identity and physical checks were reportedly performed and considered satisfactory. The transport was carried out by a Latvian transporter and registered in the EU Trade Control and Expert System (TRACES) with a Common Health Entry Document (CHED). According to the available information, four dogs from this shipment on 21 November 2025 were sent to Austria, Belgium, France and Switzerland, respectively. As part of the epidemiological investigation, the other 19 animals (17 dogs, 2 cats) from the same shipment were destined for a location in Germany. TRACES analyses also revealed that two shipments from the same Russian animal welfare organisation, carried out by two German transporters, crossed the Belarusian-Latvian border at around the same time on the evening of 20 November and the night of 21 November 2026. As there was a suspicion that the animals from all three transports may have been in contact with each other, either at the place of origin or at the border control post, the animals from the other two transports (32 dogs and 22 cats) were also traced in Germany by the competent veterinary authorities.

**Figure 2:**
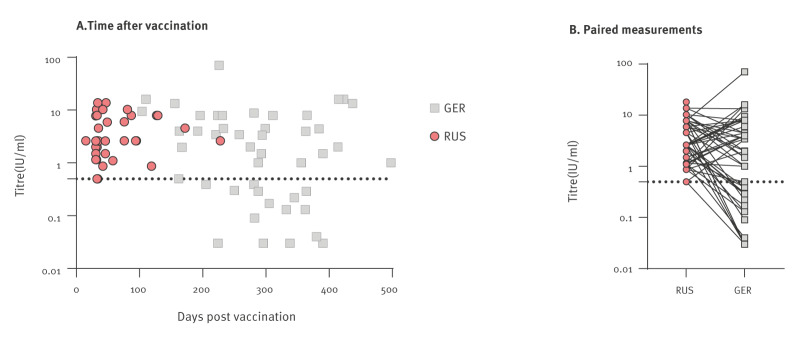
Rabies virus-neutralising antibody titres (IU/ml) in serum samples from dogs and cats measured in laboratories in Russia before entry into the European Union and in Germany following clinical investigations, March 2026

GER: Germany; RUS: Russia.

Follow-ups of all three transports revealed additional inconsistencies in the documentation in nine cases, including six where the same falsified vaccine label was used. Repeated serological testing revealed titres > 0.5 IU/ml in 33 animals, while in 16 animals, titres < 0.5 IU/ml were measured (Figure 2). While fraudulent paperwork cannot be excluded, however, temporal antibody titre dynamics and inherent inter-laboratory variability may also be considered. Interestingly, in a previous study, imported dogs from Romania and Russia also showed significantly lower titres as compared with dogs vaccinated in Finland [8].

Panel A. Serum samples from 30 dogs and 14 cats were included. One sample per animal.

Panel B. Paired measurements for samples from individual animals (n = 49) in a direct comparison between laboratories.

The dashed line indicates the commonly accepted protective threshold (0.5 IU/ml). Fourteen sera from pets that received booster vaccinations after blood sampling were excluded.

According to the TRACES documentation, a Russian animal welfare organisation near Moscow was listed as the place of origin for all shipped animals. However, phylogenetic analyses suggest that the virus, and most likely the dog itself, originated from south-central regions of Russia.

## Public health measures

Contact tracing for the rabid dog identified eight exposed persons, six persons with World Health Organization (WHO) category I exposure and two with category II exposure. All identified contact persons received post-exposure prophylaxis (PEP), although persons with category I exposure were advised that PEP was not indicated according to the WHO recommendations [9]. On the veterinary public health side, within the household, only one unvaccinated cat had direct contact with the rabid dog and was subsequently euthanised. Control measures for the other animals included in the transports were subsequently applied, including quarantine, serological testing and, if necessary, revaccination.

## Discussion

Violations of the regulatory framework have repeatedly resulted in the importation of rabies-infected animals into the EU, necessitating extensive public health responses, including contact tracing and administration of PEP [10-14]. The high demand for puppies within the EU is incentivising partly illegal imports [15]. In response, the EU launched an initiative to counter non-compliant movement and illegal trade of pets [16]. In the present case, animal welfare organisations were involved in the transport of the rabid dog and other animals into the EU. According to findings of the EU enforcement action [17], a significant number of traders abuse the EU legislation on non-commercial transport of animals to disguise their real commercial activities and seek advantages from limited controls and tax elusion. Some shelters or animal welfare associations were also found abusing their status to illegally breed or trade dogs through online advertisements and sales. Many occurrences of forged or incomplete official documents, fake or incorrect rabies vaccination information and of animals being sold while underage were noticed [17].

Vaccination and serological testing remain key measures for mitigating rabies risks associated with animal imports [3-5]. Although the presented documentations indicated an adequate antibody titre following vaccination, discrepancies observed during subsequent re-testing raise concerns. Methodological explanations, including issues related to reagents, appear unlikely in the context of accredited testing procedures. Instead, falsification or fraud at various stages of the importation process—such as the submission of mislabelled serum samples—must also be considered.

## Conclusion

This case highlights the ongoing risk posed by irregular or falsified documentation in the transboundary movement of companion animals. Despite documented vaccination and serological testing, the discrepancies identified during re-testing raise serious concerns regarding the reliability and integrity of import procedures. Given the severe public health implications associated with the introduction of rabies, and in light of the evidence presented here, strict oversight of pet imports is warranted. Furthermore, a moratorium on the importation of pets into the EU from high-risk countries appears justified.

## Data Availability

All data generated or analysed during this study are included in this published article and Supplementary material.

## Supplementary Data

411000 Supplementary Material
