# Supplementary material for: Rabies in a dog commercially imported into Germany from Russia, 2026
Source: Euro Surveill. 2026 May 14;31(19):2600372. doi: 10.2807/1560-7917.ES.2026.31.19.2600372 (PMC13179480; doi:10.2807/1560-7917.ES.2026.31.19.2600372)
Supplement: Supplementary Material [file 26-00372_FREULING_Supplement.pdf]

Supplementary material

This supplementary material is hosted by *Eurosurveillance* as supporting information alongside the article "Rabies in a dog commercially imported into Germany from Russia, 2026", on behalf of the authors, who remain responsible for the accuracy and appropriateness of the content. The same standards for ethics, copyright, attributions and permissions as for the article apply. Supplements are not edited by *Eurosurveillance* and the journal is not responsible for the maintenance of any links or email addresses provided therein.

Next-generation sequencing (NGS)

For the sample, ca. 1-2g of brain material was homogenized on an UPHO tissue homogenizer (Geneye) and 750 µl TRIzol added to 250 µl homogenate for inactivation. The TRIZol-sample mixture was processed with 200 µl chloroform followed by a ten-minute incubation at room temperature and subsequent centrifugation for ten minutes at 13.000 rounds per minute. RNA was extracted from the separated aqueous phase utilizing the RNAdvance Tissue Kit (Beckman Coulter) on a KingFisher Flex platform (Thermo Fisher Scientific) including a DNAse digestions step. The extracted and cleaned RNA was quantified on an Implen N60 Nanophotometer (Implen, Munich, Germany). For the generation of double stranded cDNA, 350 ng of RNA were processed with the Super Script™ IV First Strand Synthesis System (Thermo Fisher Scientific, Waltham, USA) followed by a second strand synthesis with the NEBNext® Ultra II Non-Directional RNA Second Strand Synthesis Modul (E6111L, New England Biolabs, Ipswich, USA) according to the manufactures instructions. The generated double stranded cDNA was quantified on an Implen N60 and 200 ng DNA each were processed with the Rapid Barcoding Kit (SQK-RBK114; Oxford Nanopore Technologies, Oxford, UK) to obtain two libraries. Sequencing was conducted on an R10.4.1 PromethION flow cell (FLO-PRO114M; Oxford Nanopore Technologies) on a PromethION 2 solo device utilizing the super-accurate basecalling mode (400bps) provided in Dorado (v7.9.8).

Table S1) Sequencing details and results

Sample ID	Library number	ENA project number	Sequencing platform	Read number (total)*	Read number (RABV)**	Proportion RABV Reads [%]
55460	7512/7513	PRJEB112407	PromethION 2 solo	7,960,116	46,042	0,58

* Raw reads trimmed with BBDuk (v. 38.84)

** Mapping against validated RABV contig lib07512_RABV_complete_genome

Sequencing data analysis

Data generated for both libraries were trimmed using BBDuk (v. 38.18) and the following parameters: qtrim = rl, trimq = 6, minlength = 50, ordered = t, qin = 33. Trimmed reads were mapped against the RABV reference sequence NC_001542 using Minimap2 (v. 2.24, preset parameter: -x map-ont) resulting in a preliminary RABV contig that was used as reference in a second mapping round. All alignments were manually inspected in Geneious Prime (v. 2025.1.3, Dotmatics) and corrected if necessary.

The so obtained complete genomes were aligned with publicly available RABV reference sequences (see Supplementary Table S1) (predefined outgroup) utilizing the multiple sequence alignment software MAFFT v7.490 [1,2] (automatic method selection). Phylogenetic analyses were performed with IQ-Tree v3.0.1 including best fit model selection by ModelFinder [3] and 100.000 ultrafast bootstraps. Tree annotation was realized with iTOL [4] v7.2.2 (European Molecular Biology Laboratory).

Table S1 Reference sequences selected from public databases for the genetic classification

Accession	Accession	Accession	Accession	Accession
**NC_001542**	OP477393	OP477368	MG458310	OL449092
**LT909526**	OP477384	ON366706	KX148133	OM203138
**KX148189**	OP477388	ON366709	KX148121	OL515141
**KX148190**	KT728349	ON366710	KX148119	OL515139
**KY860612**	KT728348	OP477377	KX148120	OL515140
**KY860609**	OP477371	OP477386	LT909547	OL449093
**KX148161**	OP477378	OP477383	KX148124	OL449095
**MK598340**	OP477389	OP477391	KX148123	OL515137
**KX148160**	MK760707	OP477372	KX148122	OL515138
**KY765901**	MK760731	OP477374	MG458306	MK598368
**OP477380**	MK760753	OP477390	OM542187	MK598362
**LN879480**	KX148159	OP477370	OM542201	MK598352
**OQ603652**	MK760712	OP477379	OM542194	MK598356
**OQ603640**	MK760734	OP477369	OM542203	MK598354
**KM016899**	MK760723	KX148143	OM542202	MK598372
**KC252633**	MK760706	MK598398	OM542193	MK598381
**PP760425**	MK760700	KX148141	OM542199	MK598393
**KY649620**	MK760755	KX148142	OM542200	MK598380
**MH267792**	MK760744	MG458311	OM542196	MK598385
**MF172976**	JQ944705	KX148140	OM542197	OM021440
**OP477381**	MF197741	KX148139	OM542198	OM203141
**OP477392**	KC595281	KX148136	KX148153	MW177593
**OP477373**	KC595282	KX148138	LT909532	OM542185
**OP477382**	KC595283	KX148135	KX148150	OM542186
**OP477376**	KC595280	LN879481	KX148148	OM542192
**OP477387**	KP997032	EU293115	LT909537	OM542189
**OP477385**	ON366707	KX148134	KF154997	OM542190
**OP477375**	ON366708	KX148132	LT909538	OM542191
**OM542204**	OQ544456	PV178704	MW177595	OL515150
**OQ544454**	OQ544455	OL440112		

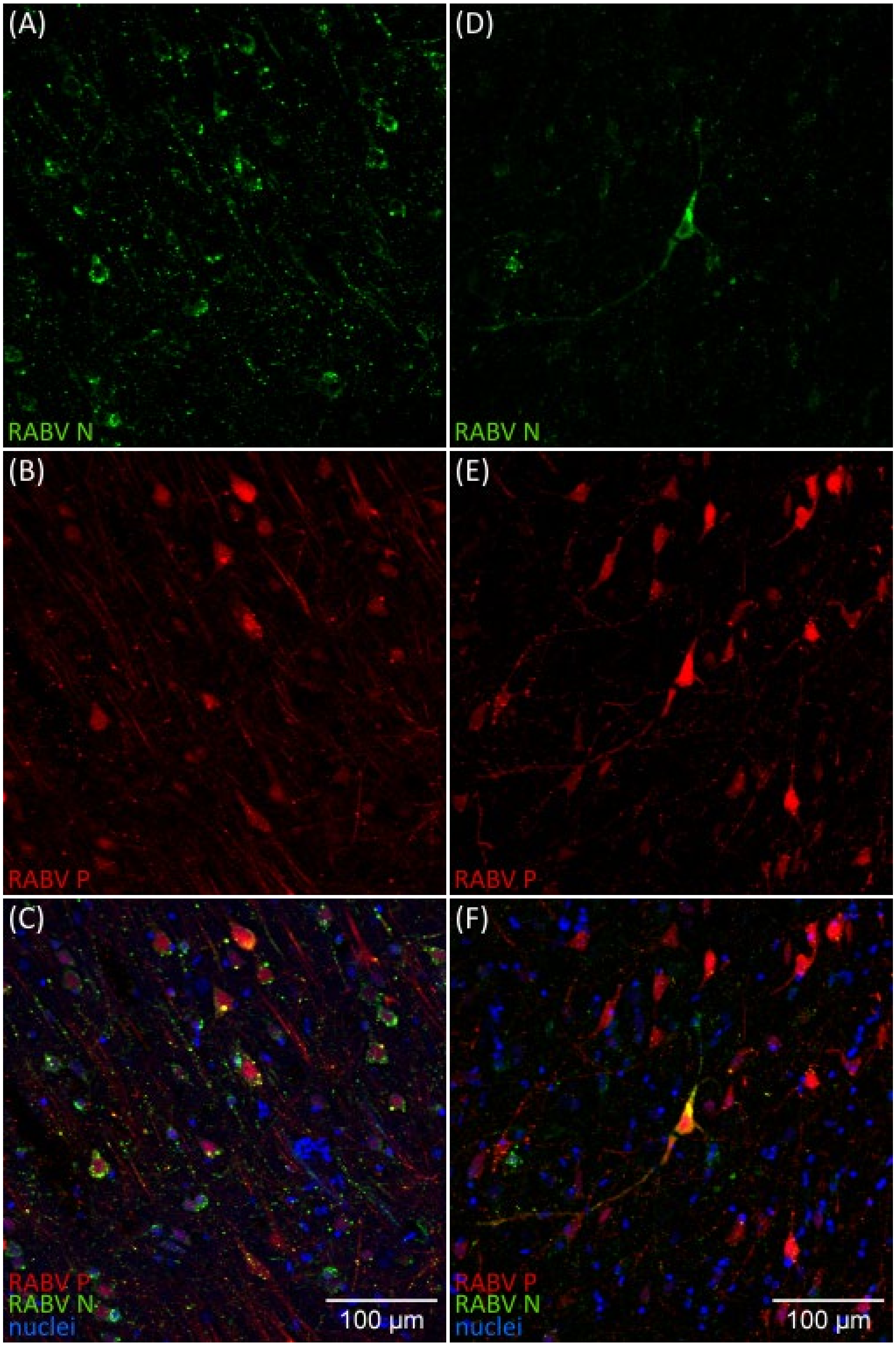

Figure S1: Confocal laser scanning microscopy images of brain tissue. (A-C) Cerebral tissue maximum z-projection of 16 optical slices, step size 1,04 µm. (D-F) Cerebellar tissue maximum z-projection of 17 optical slices, step size 0,2 µm. Viral proteins are shown as (A, D) RABV nucleoprotein (RABV N, green) and (B, E) phosphoprotein (RABV P, red). (C, F) merged images with additional Hoechst nuclear staining (nuclei, blue).

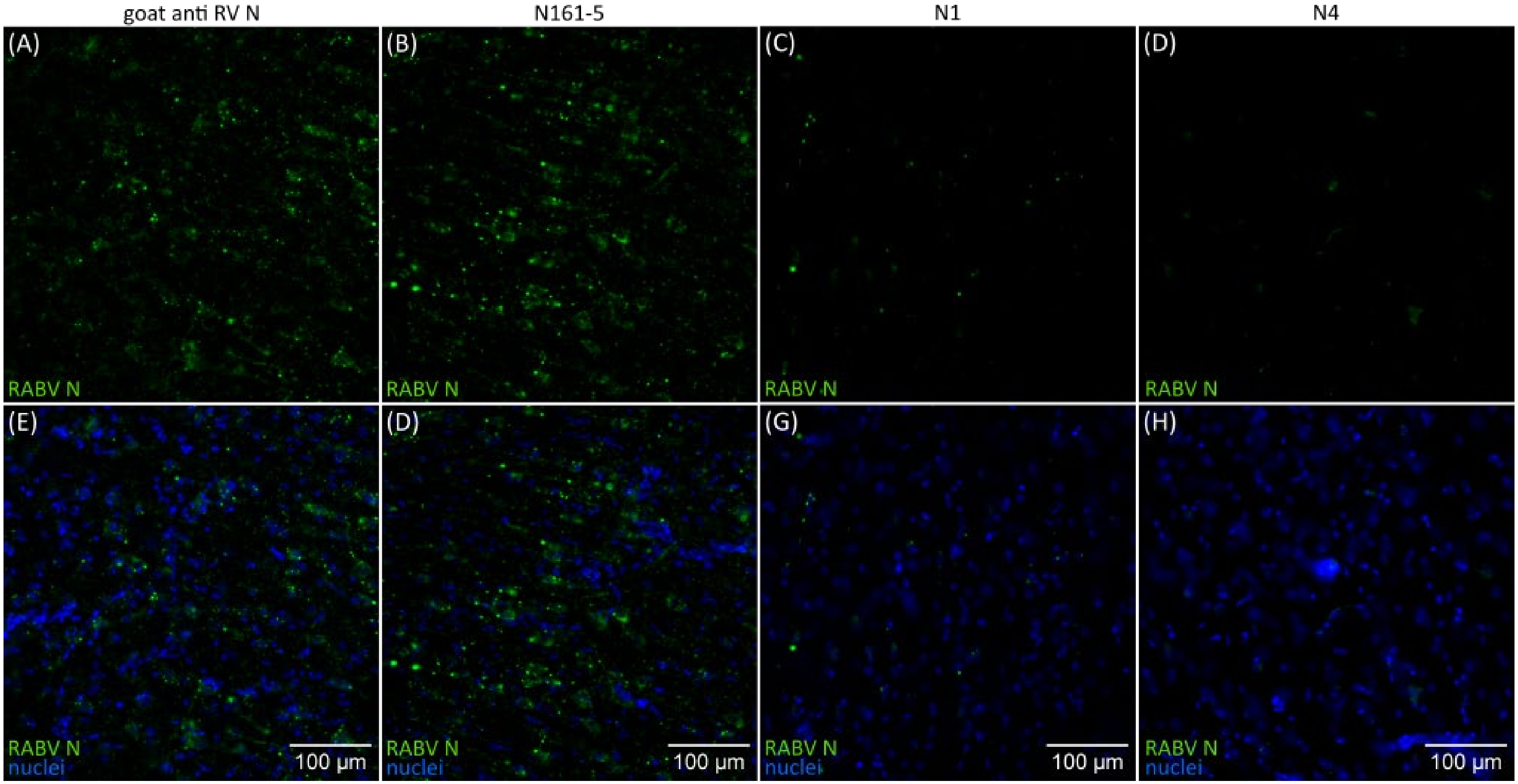

Figure S2: Fluorescence microscopy images of cerebellar tissue. (A-D) RABV nucleoprotein (RABV N, green); (E-H) merged images with additional Hoechst nuclear staining (nuclei, blue). Mouse monoclonal antibodies N1 and N4 are present in the SIFIN-conjugate.

Tissue preparation and immunolabelling

Brain tissue was fixed with 4% paraformaldehyde (PFA) in phosphate-buffered saline (PBS) and sectioned into 50 µm slices using a vibratome (VT1200S, Leica Biosystems, Germany). All subsequent steps were performed at 37°C. Samples were blocked for 24 hours (6% donkey serum/0,02% NaN3 in PBS). For RABV detection, sections were incubated for 24 hours with primary antibody diluted in 1% donkey serum/0,02% NaN3 in PBS. Samples were washed five times in PBS/0,02% NaN3, then incubated 18 hours with the secondary antibodies diluted in 1% donkey serum/0,02% NaN3 in PBS. Cell nuclei dye was subsequently incubated for one hour followed by a final series of five washes in PBS/0,02% NaN3 before mounting the sections on microscope slides using ibidi Mounting Medium (REF 50001).

Antibodies and dyes

Different antibodies were used for RABV detection: a polyclonal rabbit serum against recombinant RABV P (P160-5; 1:3000) and N (N161-5, 1:500) protein ; a polyclonal goat serum against RABV N protein (goat anti RV N, 1:400), generated by immunisation of a goat with baculovirus-expressed, purified His-tagged RABV N protein [5]; two mouse monoclonal antibodies against recombinant N protein [N1 (W239.17), undiluted; N4 (MW187.6.1), 1:30 [6].

The following secondary antibodies were used: Donkey anti-Rabbit IgG (H+L) Highly Cross-Adsorbed, Alexa Fluor™ 568 (Invitrogen Antibodies; A10042; RRID: AB_2534017; 1:500), Donkey anti-Goat IgG (H+L) Cross-Adsorbed, Alexa Fluor™ 568 (Invitrogen Antibodies; A-11057; RRID: AB_2534104; 1:500), Donkey anti-Rabbit IgG (H+L) Highly Cross-Adsorbed, Alexa Fluor™ 647 (Invitrogen Antibodies; A-31573; RRID: AB_2536183; 1:500), Donkey anti-Mouse IgG (H+L) Highly Cross-Adsorbed, Alexa Fluor™ 568 (Invitrogen Antibodies; A10037; RRID: AB_11180865; 1:500). Cell nuclei were stained using Hoechst 33342 (Invitrogen; H3570; 1:20.000).

Fluorescence microscopy, Confocal laser scanning microscopy and image processing

Image stacks of the cerebellum (step size: 1,04 µm) and cerebrum (step size: 0,2 µm) were acquired using a Leica Stellaris 8 microscope equipped with a HC PL APO 20x/0.75 IMM CORR CS2 objective using the Leica application suite software (LAS X 4.9.0.30221). Widefield fluorescence images were acquired with a Leica DMi8 Inverted Microscope equipped with a HC PL FLUOTAR L 20x/0.40 DRY objective under Leica application suite software (LAS X 3.7.4.23463). All images were processed in ImageJ (2.16.0/1.54p).

References

1. Katoh, K.; Misawa, K.; Kuma, K.; Miyata, T. MAFFT: a novel method for rapid multiple sequence alignment based on fast Fourier transform. *Nucleic Acids Res* **2002**, *30*, 3059–3066, doi:10.1093/nar/gkf436.
2. Katoh, K.; Standley, D.M. MAFFT multiple sequence alignment software version 7: improvements in performance and usability. *Mol Biol Evol* **2013**, *30*, 772–780, doi:10.1093/molbev/mst010.
3. Kalyaanamoorthy, S.; Minh, B.Q.; Wong, T.K.F.; Haeseler, A. von; Jermiin, L.S. ModelFinder: fast model selection for accurate phylogenetic estimates. *Nat Methods* **2017**, *14*, 587–589, doi:10.1038/nmeth.4285.
4. Ciccarelli, F.D.; Doerks, T.; Mering, C. von; Creevey, C.J.; Snel, B.; Bork, P. Toward automatic reconstruction of a highly resolved tree of life. *Science* **2006**, *311*, 1283–1287, doi:10.1126/science.1123061.
5. Orbanz, J.; Finke, S. Generation of recombinant European bat lyssavirus type 1 and inter-genotypic compatibility of lyssavirus genotype 1 and 5 antigenome promoters. *Arch Virol* **2010**, *155*, 1631–1641.
6. Cox, J.H.; Schneider, L.G.; Müller, W.W. Eine Antigenvariante der Fuchstollwut in Europa. *Tieraerztl Umsch* **1992**, *47*, 824–828.
